# AI-Assisted Physics-Informed Predictions of Degradation Behavior of Polymeric Anion Exchange Membranes

**DOI:** 10.1021/acs.jpcb.5c07063

**Published:** 2026-01-27

**Authors:** William Schertzer, Mohammed Al Otmi, Janani Sampath, Ryan P. Lively, Rampi Ramprasad

**Affiliations:** † School of Materials Science and Engineering, 1372 Georgia Institute of Technology, 771 Ferst Drive, J. Erskine Love Building, Atlanta, Georgia 30332-0245, United States; ‡ Department of Chemical Engineering, 3463 University of Florida, 1006 Center Drive, Gainesville, Florida 32611, United States; § School of Chemical and Biomolecular Engineering, 1372 Georgia Institute of Technology, 311 Ferst Drive NW, Ford Environmental Science & Technology Building, Atlanta, Georgia 30332-0100, United States

## Abstract

The global transition to hydrogen-based energy infrastructures faces significant hurdles. Chief among these are the high costs and sustainability issues associated with acid–based proton exchange membrane fuel cells. Anion exchange membrane (AEM) fuel cells offer promising cost-effective alternatives, yet their widespread adoption is limited by rapid degradation in alkaline environments. Here, we develop a framework that integrates mechanistic insights with machine learning, enabling the identification of generalized degradation behavior across diverse polymeric AEM chemistries and operating conditions. Our model successfully predicts long-term hydroxide conductivity degradation (up to 10,000 h) from minimal early time experimental data. This capability significantly reduces experimental burdens and may expedite the design of high-performance, durable AEM materials.

## Graphical abstract

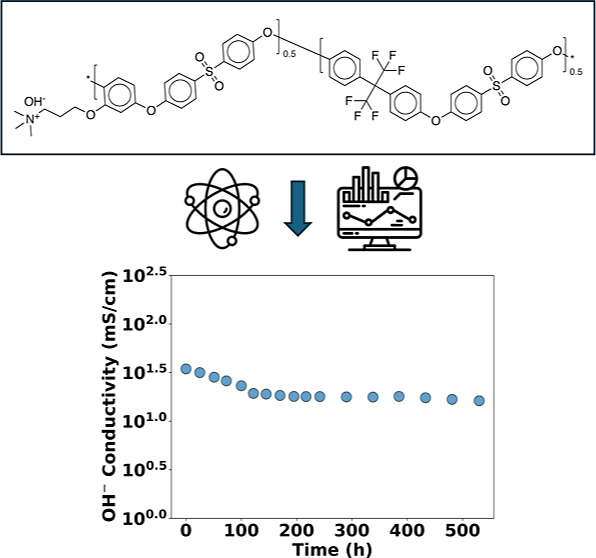

## Introduction

Increasing global demand for clean energy has spurred widespread interest in the development of cost-effective and efficient fuel cell technology.^1,2^ Although proton exchange membrane (PEM) fuel cells have received the majority of research attention to date, their reliance on costly and environmentally persistent perfluorinated polymers (e.g., Nafion) and platinum-based catalysts significantly limits their scalability and sustainability.^1,3^

Anion exchange membrane (AEM) fuel cells operate under alkaline conditions and conduct hydroxide ions with faster reaction kinetics compared to their PEM counterparts, enabling the use of inexpensive, fluorine-free hydrocarbon-based polymers and nonprecious metal catalysts.^4,5^ These potential cost advantages have driven significant research interest in AEMs over the past two decades, positioning them as promising alternatives to conventional acid–based fuel cells.^5–8^ This shift not only reduces cost and environmental burden but also opens pathways for more sustainable polymer design.^8^ However, the commercialization of AEM fuel cells is still hindered by major challenges, notably the chemical and mechanical instability of AEMs in alkaline media, which leads to rapid degradation and failure far before the 20,000–25,000 h target operational lifetime set by the U.S. Department of Energy.^9^ This challenge is coupled with the need for membranes with high hydroxide conductivity, e.g., greater than 100 mS/cm.^4^ Unfortunately, chemical durability and ionic conductivity are material properties that are often in conflict.^10^

A significant body of research has focused on improving individual aspects of AEM performance—such as enhancing ion exchange capacity (IEC), reducing water uptake (WU) and swelling ratio (SR), and improving hydroxide conductivity.^1,8,11^ Our previous work contributed to this area by leveraging machine learning and atomistic models learning to predict these static properties and identify fluorine-free AEM candidates that strike a balance between performance and stability.^12–14^ Yet, while such models capture initial performance, they offer limited insight into the long-term degradation behavior that ultimately governs membrane viability in real-world applications, and they fail to capture the interplay between these individual aspects as they relate to long-term durability.^15–17^

Recent advances in machine learning research have led to the development of various types of robust algorithms suitable for many science and engineering applications.^18,19^ In prior work, we applied Gaussian process regression (GPR) to assess the extrapolative capability of ML models for AEMs and to quantify the chemical diversity of the available data set.^13^ That analysis revealed that the limited diversity of reported chemistries imposes inherent constraints on the accuracy of predictions for entirely novel formulations. Building on this insight, the present study shifts focus toward uncovering trends within the existing chemical space using a Physics-Enforced Neural Network (PENN).

In this study, we extend our informatics-based approach to address a critical missing dimension in AEM research: time-dependent degradation. Specifically, we focus on the evolution of hydroxide conductivity under prolonged alkaline exposure, a key indicator of chemical and structural breakdown in AEMs.^10,15^ Although prior studies have investigated the degradation of specific AEMs by introducing structural modifications (e.g., flexible spacers, cross-linking, branching, or inorganic additives^1,4,5,8^ ), these efforts have largely remained fragmented. They often focus on narrow design variations and isolated degradation mechanisms, making it difficult to generalize findings across the broader AEM landscape.

To address this gap, we have created a comprehensive database of time-resolved hydroxide conductivity measurements from the literature. The database encompasses a wide diversity of polymer backbones, cationic groups, solvents, additives, temperatures, and relative humidities. Upon observing time-dependent hydroxide conductivity trends across a breadth of chemistries and environmental conditions, we identified a consistent empirical relationship for the degradation of hydroxide conductivity in any AEM system exposed to alkaline media for prolonged periods. eq 1 is proposed to describe the degradation of the hydroxide conductivity of AEMs.

1 $$\log\sigma \left(t\right)=\log\sigma_{\infty }+\frac{\log\sigma_{0}-\log\sigma_{\infty }}{1+{\left(\frac{t}{t_{0}}\right)}^{\alpha }}$$

In this equation, σ_0_ represents the initial hydroxide conductivity at time = 0, while σ_∞_ is the limiting conductivity at long times or under equilibrium conditions. The parameter *t*_0_ is a characteristic time scale that governs the halfway drop-off point from log σ_0_ to log σ_∞_, and α is a shape parameter that determines the steepness of the decay curve. This equation captures the time-dependent degradation of conductivity due to complex chemical processes under alkaline conditions, reflecting the initial performance, long-term stability, and rate of performance decay of AEMs.

We then introduce a PENN framework designed to uncover universal degradation trends from this heterogeneous data set by predicting the four parameters (σ_0_, σ_∞_, *t*_0_, α) for each AEM sample. Figure 1 depicts the PENN architecture employed here: polymer genome fingerprints^20^ are concatenated with environmental variables and passed through a multilayer perceptron (MLP) to predict the four output parameters of eq 1. These predicted parameters are scaled to physically reasonable ranges and then passed to the loss function along with the measurement time and ground truth conductivity value for each sample to achieve model training.

**Figure 1:**
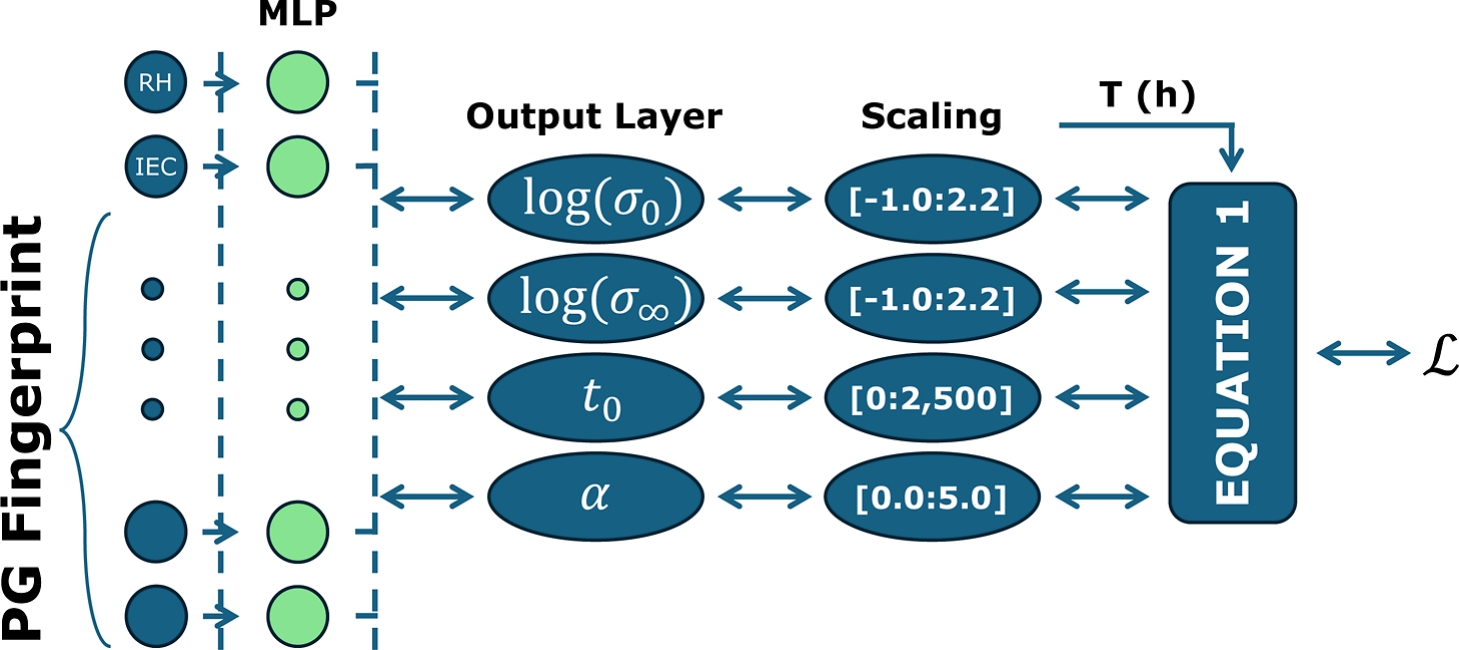
Schematic of the PENN architecture. Polymer genome fingerprints and environmental features are passed through a multilayer perceptron (MLP), which predicts four physically meaningful degradation parameters: initial conductivity σ_0_, limiting conductivity σ_∞_, characteristic time *t*_0_, and decay shape parameter α. These parameters are then plugged into a mechanistic degradation equation and compared to experimental time series to guide training via a physics-informed loss function.

AEM degradation is driven by complex coupled chemical processes—such as β-elimination, nucleophilic substitution, and polymer backbone scission—that occur under alkaline conditions and are influenced by the chemistry of the polymer, the IEC, the degree of cross-linking, the presence of stabilizing or destabilizing additives, and the operating environment (e.g., temperature and relative humidity).^1,10^ These intertwined effects make it difficult to isolate causal relationships using traditional empirical studies and underscore the need for a unified framework capable of modeling long-term behavior across a chemically diverse set of AEMs.^5^

If eq 1 is true, then appropriately normalizing the degradation curves would reveal a universal behavior across multiple samples. eq 2 shows the normalized degradation equation, in which the degradation curves of all systems may collapse onto a single master curve, suggesting a universal degradation behavior that transcends specific chemistries and environmental conditions.

$$\begin{array}{c} \log\mathrm{\sigma ̂}\left(t\right)=\frac{1}{1+\mathrm{t̂}},\\ \mathrm{where}\;\log\mathrm{\sigma ̂}=\frac{\log\sigma -\log\sigma_{\infty }}{\log\sigma_{0}-\log\sigma_{\infty }}\;\text{and}\;\mathrm{t̂}={\left[\frac{t}{t_{0}}\right]}^{\alpha } \end{array}$$ 2

By passing the predicted parameters and measurement time for each sample through eq 2 and visually comparing the σ̂ vs t̂ curves, we show that there is indeed a predictable set of parameters for each sample such that the normalized predicted and observed hydroxide conductivity are in agreement across the observed range of AEM formulations.

Going further toward helping make efficient engineering decisions, we demonstrate the ability of our PENN framework to distinguish between different degradation modes and to extrapolate long-term degradation behavior from short-term data. A comparison of PENN vs baseline NN and GPR models (in which eq 1 is not enforced) shows that our proposed method excels at identifying samples that exhibit bimodal degradation patterns (rapid initial degradation followed by smooth, gradual degradation), where NN overfits (predicting nonphysical increasing trends) and GPR oversimplifies (predicting nonphysical smooth degradation trends). We achieve accurate predictions of hydroxide conductivity at several thousands of hours using only a few hundred hours of early time measurements, and we show the jump in forecasting predictions when using PENN compared to NN and GPR. These capabilities have the potential to drastically reduce the experimental burden associated with long-term stability testing.

## Methods and Materials

### Data Set

The training data set used in this study contains over 5200 data points manually extracted from tables and figures in academic articles with the help of the WebPlotDigitizer tool.^21^ The data set, along with the associated DOI of each entry, is publicly available on the polyVERSE GitHub https://github.com/Ramprasad-Group/polyVERSE/tree/main/Other/Conductivity_anionic_aging. Both static and time-resolved property measurements are recorded, with over 2,200 unique hydroxide conductivity data points, each corresponding to a distinct AEM system. The property values were recorded at time points spanning from initial synthesis and preparation (*t* = 0 h) to complete membrane failure (*t* ≥ 10,000 h) under various experimental conditions. The data set contains 112 unique profiles of time-resolved hydroxide conductivity measurements of AEM formulations. In many of these profiles, we observed an initial rapid increase in hydroxide conductivity prior to its eventual decay. This “waking up” effect is likely due to membrane hydration. To ensure consistent model training and reflect the true onset of degradation, we shifted the time axis such that *t* = 0 corresponds to the point of maximum conductivity for each sample. A summary of the number of data points available for each property, along with the percentage of each property in the data set and the percentage of time-dependent data, is provided in Table 1.

**Table 1:** Summary of Dataset Properties, Including the Percentage of Dataset for Each Property, the Percentage of That Property with Time-Dependent Data, and the Number of Data Points for Each Property

property	number of data points	% of data set	% time dependent
OH^–^ cond. (mS/cm)	2,229	40.54	50.51
ion exchange capacity (meq/g)	1,485	27.53	30.58
water uptake (wt %)	627	11.40	3.03
swelling ratio (%)	521	9.53	2.20
tensile strength (MPa)	171	3.11	10.52
elongation at break (%)	163	2.96	8.30
young’s modulus (MPa)	73	1.32	0
total	5,269	100	33.23

In preparation for machine learning analysis, each profile was annotated with its unique combination of chemical and environmental descriptors, reflecting both polymer composition and test conditions. These include:

1. Monomer structure: represented as SMILES strings (SMILES1-SMILES3) for each monomeric repeat unit in the copolymer.
2. Monomer composition: mole fractions (c1-c3) indicating the proportion of each monomer in the statistical copolymer backbone.
3. Theoretical ion exchange capacity (IEC): the number of active ion exchange sites per polymer repeat unit (reported in meq/g), calculated from polymer structure and composition, as described in our previous contribution.^13^
4. Relative humidity (RH): the ambient humidity (reported in percent) during conductivity measurement.
5. Stability test temperature: the temperature (reported in °C) at which the degradation experiment was conducted.
6. Measurement temperature: the temperature (reported in °C) at which the conductivity measurement was recorded.
7. Solvent type and concentration: the identity of the solvent used during degradation testing (e.g., KOH, NaOH) and its reported concentration (reported as molarity [mol/L]).
8. Additive type and concentration: the identity of the additive(s) used during degradation testing (e.g., stabilizers, cross-linkers, inorganic fillers) and the corresponding concentration (reported in wt %).
9. Time: the amount of time (reported in hours) that the sample has been submerged in a particular solvent.

In addition to conductivity, the data set also includes both static and time-resolved measurements for other key AEM properties (ion exchange capacity, water uptake, swelling ratio, tensile strength, elongation at break, and Young’s modulus), all of which have been discussed in our previous contribution. Although this work focuses exclusively on modeling the time evolution of hydroxide conductivity, these additional properties offer valuable insight into mechanical and transport degradation behavior. In the future, a multitask framework that jointly models the degradation of conductivity, swelling, and mechanical performance will be explored to enable holistic lifetime prediction of AEMs given sparse time-resolved property data.

### Feature Engineering

Chemical features were extracted from each sample using the co-polymer genome fingerprinting scheme, which encodes the hierarchical structure of copolymers and has been shown to accurately predict a wide range of polymer properties, including hydroxide conductivity, water uptake, and swelling ratio.^20,22^ Each monomeric repeat unit is converted into a chemically informed descriptor vector, capturing atomic, structural, and electronic features. These vectors are combined into a single polymer fingerprint via a composition-weighted linear combination, reflecting the molar ratios of monomers in the copolymer backbone.

To this base representation, we append experimentally relevant environmental descriptors, including IEC, RH, and temperature, as described in eqs 1–3 of our prior work.^13^ In the present study focused on modeling time-resolved degradation, we extend this fingerprinting framework to include new physicochemical and environmental features that influence degradation under alkaline conditions. These include:

1. Stability test temperature, reflecting the thermal environment during degradation experiments.
2. Solvent concentration, capturing the identity and strength of the alkaline medium (e.g., KOH, NaOH).
3. Additive concentration, encompassing stabilizers, cross-linkers, or inorganic fillers added to enhance stability.

Continuous-valued concentration features of each solvent and additive are used to encode the presence and relative amount of each component in the sample. The final feature vector, comprising polymer structure, environmental descriptors, and processing conditions, is fully normalized on a scale of [0:1] to ensure numerical stability and effective training. Although this work employed the co-polymer genome fingerprinting scheme, the key conclusions of this work do not depend on the use of a specific fingerprint. Rather, the machine learning framework is agnostic to the particular polymer descriptor choice and learns a mapping from any sufficiently expressive polymer representation, combined with environmental variables.

### Machine Learning Framework

Three machine learning (ML) models were implemented to model time-dependent degradation in AEMs: Gaussian process regression (GPR), a classic neural network (NN) and a physics-enforced neural network (PENN). GPR and NN served as nonphysics baselines, while PENN incorporated physical constraints into its architecture to capture degradation dynamics.

### GPR and NN: Non-Physics Baselines

GPR was implemented as a nonparametric Bayesian regression method capable of producing both point predictions and associated uncertainty estimates.^23,24^ Hydroxide conductivity was predicted directly from a feature vector including polymer fingerprints, environmental variables, additive descriptors, and time as an explicit input feature. A composite kernel combining a radial basis function and white noise was employed to capture both smooth nonlinear relationships and experimental noise. GPR models were trained using Scikit-learn^25^ with 5-fold cross-validation across five random seeds. Model performance was averaged over the folds and seeds to ensure statistical robustness.

NN was implemented as an additional baseline to see if a simple neural network approach could mitigate the issues with GPR, or if a physics-enforced architecture was necessary for this application. The NN was implemented as a fully connected feedforward neural network model using PyTorch.^26^ The network comprised an input layer matching the dimension of the fingerprinted feature vector (chemical descriptors, time, etc), three hidden layers with nonlinear activation functions and dropout layers, and an output layer predicting the log of conductivity. Hyperparameter optimization was performed using Optuna,^27^ which employed Bayesian optimization to explore.

- learning rate (1 × 10^–4^–1 × 10^–3^),
- hidden layer sizes (Layer 1:512–1024 units; Layer 2:128–512 units; Layer 3:64–128 units),
- dropout rates (0.1–0.5 for all layers).

The final model configuration corresponded to the hyperparameter set yielding the lowest training loss. Models were trained using the Adam optimizer with a learning rate scheduler that reduced the learning rate by a factor of 0.5 after 100 epochs without validation loss improvement, with a lower bound of 1 × 10^–6^. Training was terminated using an early stopping criterion after 200 epochs without improvement in validation loss. Loss was defined as the mean squared error between the predicted and true conductivity.

### PENN: Physics-Enforced Neural Network

The PENN model was implemented similarly to the NN model described above, with a few key differences. As illustrated in Figure 1, instead of including time as an input feature and directly predicting conductivity, the architecture was built such that the final layer was a vector of length four, with each component of the final vector corresponding to one of the scalar parameters of the degradation profile. These parameters were then scaled to align the predicted values with the empirically observed ranges and finally plugged into eq 1 along with each sample’s recorded time value to get a conductivity prediction. Hyperparameter optimization was again performed using Optuna, which explored the same parameter space as the NN case but with the addition of the physics weight parameter ω (0.00–0.50, step size 0.01), which is discussed in the PENN Architecture Design section.

## Results and Discussion

### Modeling Strategy for Time-Dependent Degradation

A central challenge in modeling AEM degradation is how to incorporate time-dependent behavior without losing the identity of the underlying material. A simple approach is to treat time as an input feature—concatenated alongside polymer fingerprints, environmental conditions, and additive concentrations. However, this method implicitly assumes that an AEM sample observed at different time points corresponds to entirely different materials, ignoring the continuity of its degradation trajectory. To overcome this, we adopt a more physically meaningful strategy: we decouple time from the input vector and instead inject it directly into a custom loss function that evaluates model predictions against the full temporal degradation profile. This allows the model to learn the universal degradation dynamics from the data while preserving the chemical identity. We compare these approaches by benchmarking a GPR model and a classic NN which use time as an input feature against a PENN that learns degradation behavior by embedding time into the modeling process itself.

While eq 1 defines a parametric form for degradation, the principal advantage of the PENN framework lies in how its parameters are inferred. Conventional parametric or hierarchical regression approaches typically fit degradation parameters independently for each material, requiring extensive time-resolved data per chemistry or strong external priors. In contrast, the PENN learns a shared, nonlinear mapping from polymer-genome descriptors and environmental variables to the degradation parameters themselves.

This feature-conditioned parameter inference enables physically meaningful extrapolation even when only sparse or early time data are available for a given system. Indeed, many samples in the present data set lack extended alkaline aging data beyond initial postsynthesis measurements. As demonstrated in the forecasting experiments (Figures 6 and 8), the PENN can infer long-term degradation behavior in such cases, whereas nonphysics neural networks and GPR baselines either fail to extrapolate or produce nonphysical trends. In this sense, the PENN functions as a data-efficient surrogate for mechanistic parameter estimation rather than a curve-fitting model.

### PENN Architecture Design

To address the limitations of GPR and classic NN, we implemented a PENN framework that leverages a mechanistic model of conductivity degradation. Rather than predicting conductivity directly at each time point, the PENN is trained to predict the parameters of eq 1. The neural network learns to predict σ_0_, σ_∞_, *t*_0_, and α for each sample given its feature vector. Time is not used as an input feature, but instead appears only in the loss function, where the predicted degradation curve is compared to the experimental conductivity time series. This formulation ensures that the model respects known physical behavior and enables accurate extrapolation beyond the training time window.

The loss function is defined as the mean squared error between predicted and true conductivity values after passing the four parameters and the time data for each sample in a particular batch through eq 1. Additional penalties are applied during training to enforce known physical constraints. Given a time-resolved sample, we can ascertain that the predicted σ_0_ should be greater than or equal to the first property measurement at *t* = 0. Similarly, the predicted σ_∞_ should be less than or equal to the final property measurement, where t is the largest value. These constraints are intended to modify the optimization landscape toward more physically relevant spaces. A small weighting parameter ω is used to optimize the amount of emphasis placed on these additional constraints.

eq 1 is not intended to represent a single elementary reaction mechanism, but rather an effective, coarse-grained description of conductivity decay arising from multiple concurrent degradation processes. In alkaline AEMs, mechanisms such as β-elimination, nucleophilic substitution, and polymer backbone scission each contribute to a progressive loss of ion-conducting functionality. Although mechanistically distinct, these processes share a common macroscopic consequence: the gradual disruption and isolation of connected ionic transport pathways.

As degradation progresses, the diminishing availability of intact cationic sites and percolated water-rich domains naturally leads to nonlinear saturation behavior, characterized by rapid early performance loss followed by slower, asymptotic decay. Such behavior is well captured by sigmoidal or logistic-like decay forms in transport properties. While more elaborate composite kinetic models could in principle capture additional mechanistic detail, the heterogeneous and limited nature of the available data set precludes unique identification of multiple sequential rate constants. eq 1 therefore represents the simplest empirically consistent functional form that robustly captures degradation behavior across thousands of measurements.

### Comparison of GPR, NN and PENN Performance on Training Data

We begin by comparing the performance of the GPR and NN baselines with PENN model on all time-resolved samples using all data for training. Figure 2 presents parity plots for all three models, where PENN achieves an overall *R* ^2^ of 0.987, slightly higher than NN’s *R* ^2^ 0.955 or GPR’s *R* ^2^ of 0.951. Although this small numerical gap might suggest similar performance, a closer inspection of degradation forecasting profiles reveals significant differences.

**Figure 2:**
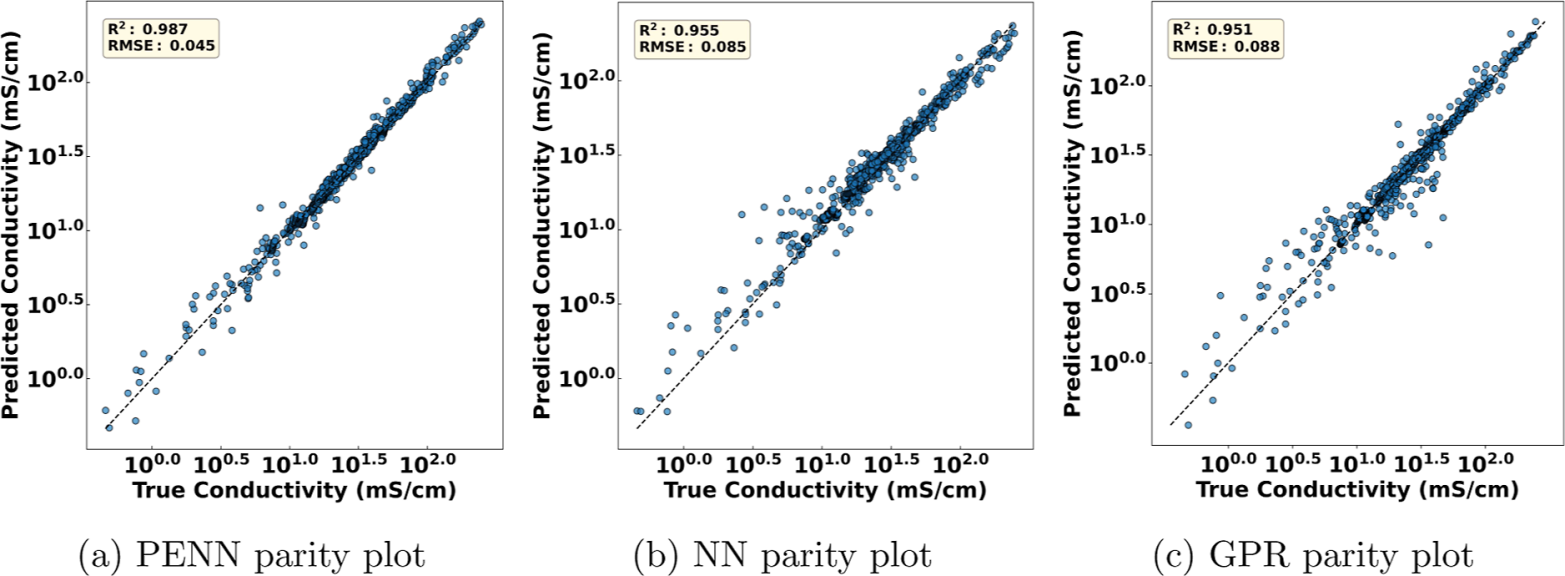
Parity plots comparing predicted versus true hydroxide conductivity across all test samples using (a) PENN, (b) NN and (c) GPR models. All models show good accuracy and consistency across the range of predicted conductivity values when using the entire data set for training.

Figure 3 shows representative degradation profiles that highlight the advantage of PENN over GPR and NN in capturing the physics of AEM degradation. By learning to predict physical parameters associated with observed degradation trends, PENN’s physics-informed architecture captures both the sharp early transition and the subsequent leveling-off phase, resulting in more accurate overall predictions and a more reliable estimate of the time required to reach stabilization. In contrast, GPR’s reliance on a stationary kernel oversmooths the early rapid changes, causing it to underestimate the initial drop and introduce slight misalignment in long-term predictions, and NN models tend to overfit the training data, providing nonphysical predictions (increasing conductivity with time).

**Figure 3:**
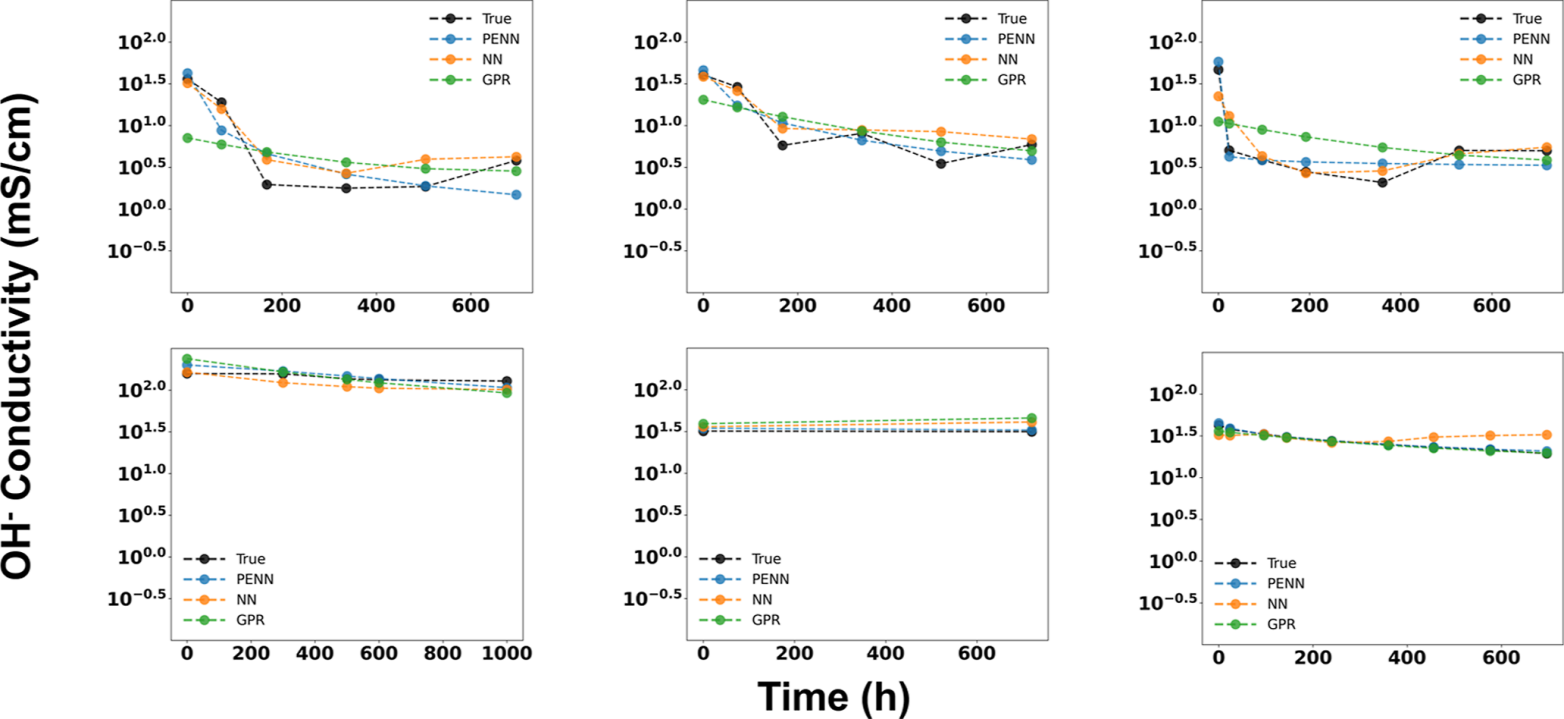
Representative degradation curves comparing PENN (blue), NN (orange) and GPR (green) predictions against experimental data (black) for six different AEM samples. Each model was trained on all available data. The top row depicts cases with more drastic degradation, while the bottom row depicts more moderate degradation profiles.

This ability to represent distinct degradation phases is critical for meaningful long-term forecasting. In real membranes, an initial period of rapid damage is often followed by a slower, stabilization-driven decay, and PENN’s mechanistic constraints allow the model to accurately capture this two-stage behavior. As a result, PENN produces predictions that are not only more accurate but also more faithful to the underlying physical processes driving membrane degradation.

Importantly, as will be demonstrated in the forecasting section, the ability to distinguish between these degradation regimes could inform the design of next-generation AEMs—enabling targeted materials development for applications where a short burst of high power output is acceptable, as well as for scenarios demanding long-term, stable performance.

### Emergence of a Universal Degradation Curve

By applying the PENN model across the full data set, we observe that the predicted degradation behavior of all samples collapses onto a single, normalized master curve when plotted using the rescaled variables defined in eq 2. This result, shown in Figure 4, confirms our hypothesis that despite the chemical and environmental diversity in the data set, degradation follows a shared empirical trajectory. This universal behavior reveals a powerful abstraction: conductivity decay in AEMs can be effectively parametrized using just four physically meaningful quantities. The ability to normalize this behavior across systems is crucial for guiding future design by establishing performance benchmarks and degradation archetypes.

**Figure 4:**
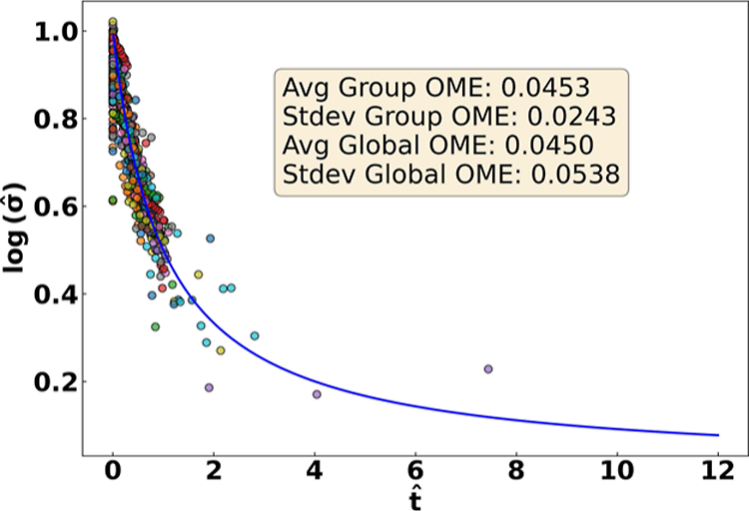
Normalized degradation behavior across all AEM samples. The PENN-predicted degradation curves collapse onto a universal master curve defined by eq 2. The blue line represents the idealized form $y=\frac{1}{1+x}$ . This agreement across chemistries and conditions reveals a shared empirical degradation mechanism and confirms the ability of the PENN to uncover universal trends.

To quantitatively assess the quality of this collapse, we evaluated the residual error of the normalized degradation curves relative to the idealized master curve defined by 2 across all samples, the normalized representation yields a global average order-of-magnitude error (OME) of 0.0450 orders of magnitude, and a standard deviation of 0.0538 orders of magnitude. When grouped by unique material–environment combinations (i.e., backbone, cation, additive, solvent, temperature, and relative humidity), the average group-level OME is 0.0453 orders of magnitude with a standard deviation of 0.0243 orders of magnitude. These low and narrowly distributed errors indicate that deviations from the master curve are small and consistent across chemically and environmentally distinct systems. Importantly, this universality does not imply identical degradation kinetics across chemistries. Rather, it emerges only after conditioning each system on its learned degradation parameters (σ_0_, σ_∞_, *t*_0_, α), which encode chemistry- and environment-specific behavior. Once normalized by these parameters, the remaining degradation trajectory exhibits a shared empirical form across diverse AEM formulations, supporting the statistical validity of the universal master curve.

We further analyze the distribution of predicted parameters σ_0_, σ_∞_, *t*_0_, and α across the data set. The histograms shown in Figure 5 highlight trends such as the clustering of α between 1.5 and 3.0, the broader variation in *t*_0_, and the bimodal distribution of σ_∞_, indicating differences in degradation kinetics between chemistries. These distributions offer insight into material design: a high α value corresponds to sharper decay after an initial stable region, whereas longer *t*_0_ implies greater resistance to degradation. Such correlations can inform rational design strategies for more durable AEMs or those with high energy burst capabilities but long-term susceptibility to degradation.

**Figure 5:**
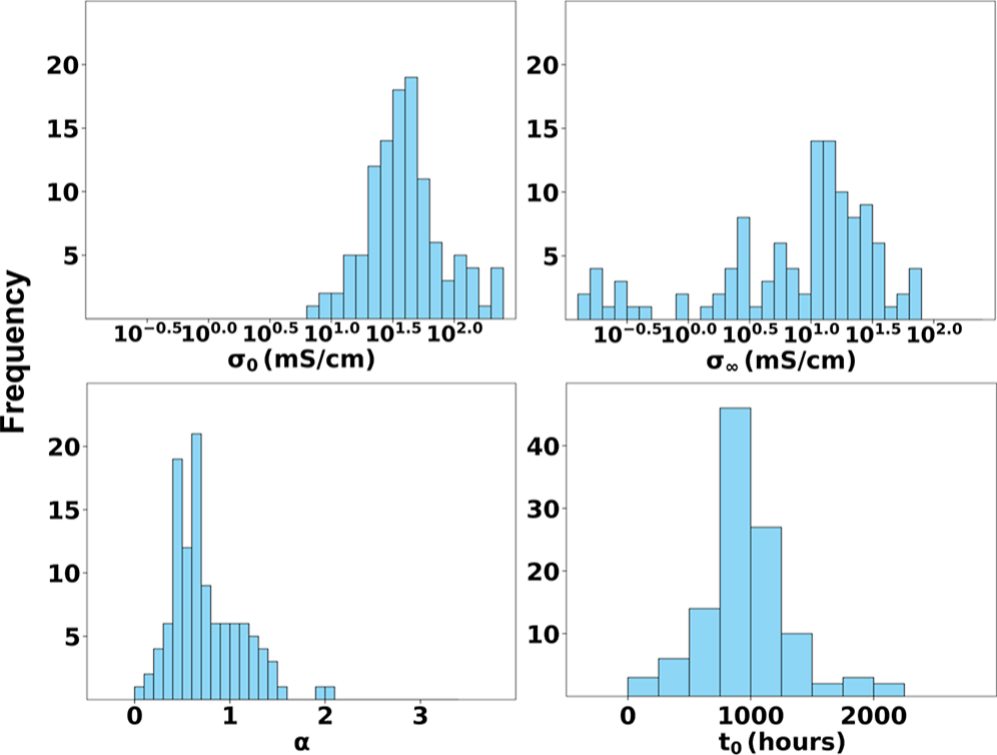
Distribution of PENN-predicted degradation parameters across all AEM samples. Histograms show the learned values of σ_0_ (top left), σ_∞_ (top right), α (bottom left), and *t*_0_ (bottom right). These distributions reflect the variability in conductivity behavior across different chemistries and testing conditions, highlighting materials with sharper or more gradual degradation.

In this scheme, models are trained on the entire data set of hydroxide conductivity profiles and predictions are made for each time-resolved sample. Then, after predicting the four parameters for each sample, they are plugged into eq 2 to compare the fit of all of the time-resolved samples at once. The goal of this approach is to identify generalizable patterns that govern the degradation of anion exchange membranes across diverse chemistries, processing conditions, and environmental exposures. By training on the complete time series data for each polymer, the model learns to capture the underlying structure of conductivity decay across thousands of degradation trajectories. For the PENN model, this enables the identification of a normalized degradation manifold that describes how conductivity evolves as a function of scaled time, independent of specific chemical details. This universal trend is particularly useful for uncovering shared degradation mechanisms and benchmarking materials against common decay baselines.

The PENN does not explicitly classify degradation as chemical or physical in origin. Instead, it infers chemistry-dependent effective degradation parameters that reflect the combined impact of all processes influencing hydroxide conductivity. Nevertheless, different mechanisms tend to manifest in distinct regions of parameter space. Physical aging phenomena—such as water redistribution, microphase densification, or counterion trapping—primarily influence early time behavior and are reflected in variations of σ_0_. In contrast, irreversible chemical degradation processes more strongly affect the long-time limit σ_∞_ and the characteristic time scale *t*_0_.

The ability of the PENN to capture two-stage degradation behavior (Figure 3) and the structured distributions of learned parameters (Figure 5) enables a phenomenological decomposition of degradation behavior that can inform mechanistic interpretation, while acknowledging that definitive attribution requires complementary experimental or spectroscopic evidence.

### Forecasting Long-Term Degradation from Early-Time Data

One of the key strengths of the PENN framework is its ability to forecast long-term degradation from short-term measurements. We implement a time-threshold validation strategy to assess the model’s ability to forecast long-term degradation from limited early time data. For each time-resolved AEM sample, we construct a model that is trained on the full data set excluding that sample’s later-time measurements. Specifically, for a given sample, only data points prior to a selected time threshold are included, while all data from other samples are retained. We repeat this procedure for every sample and for a series of thresholds: 0 h (no data from this sample is included in the training set), 50, 100, 200, 300, 400, 500, and 1000 h. Figure 6 shows PENN parity plots for the various time thresholds and indicates that with no data for a particular sample, degradation forecasting predictions are reasonable, and that even with as little as 200 h of data, PENN achieves accurate predictions of conductivity up to 10,000 h for most samples. The improvement in performance with increasing threshold demonstrates the value of early time data while also quantifying the point at which degradation forecasting becomes reliable. This capability is critical for real-world applications, where prolonged testing is often infeasible. The results from this exercise (as highlighted in the average Order of Magnitude Error (OME) vs threshold plot in Figure 7) indicate that after about 200 h of performance data there is a significant diminishing returns to collecting longer-time data to predict longer time behavior, and that the PENN models significantly outperform GPR and NN in forecasting ability with limited data, making it an ideal approach for future AEM design schemes. As reflected by the shaded regions in Figure 7, which represent ±0.25σ (one-quarter of the standard deviation) around the mean OME, the PENN model also exhibits lower variability across samples, indicating greater robustness and generalization capability.

**Figure 6:**
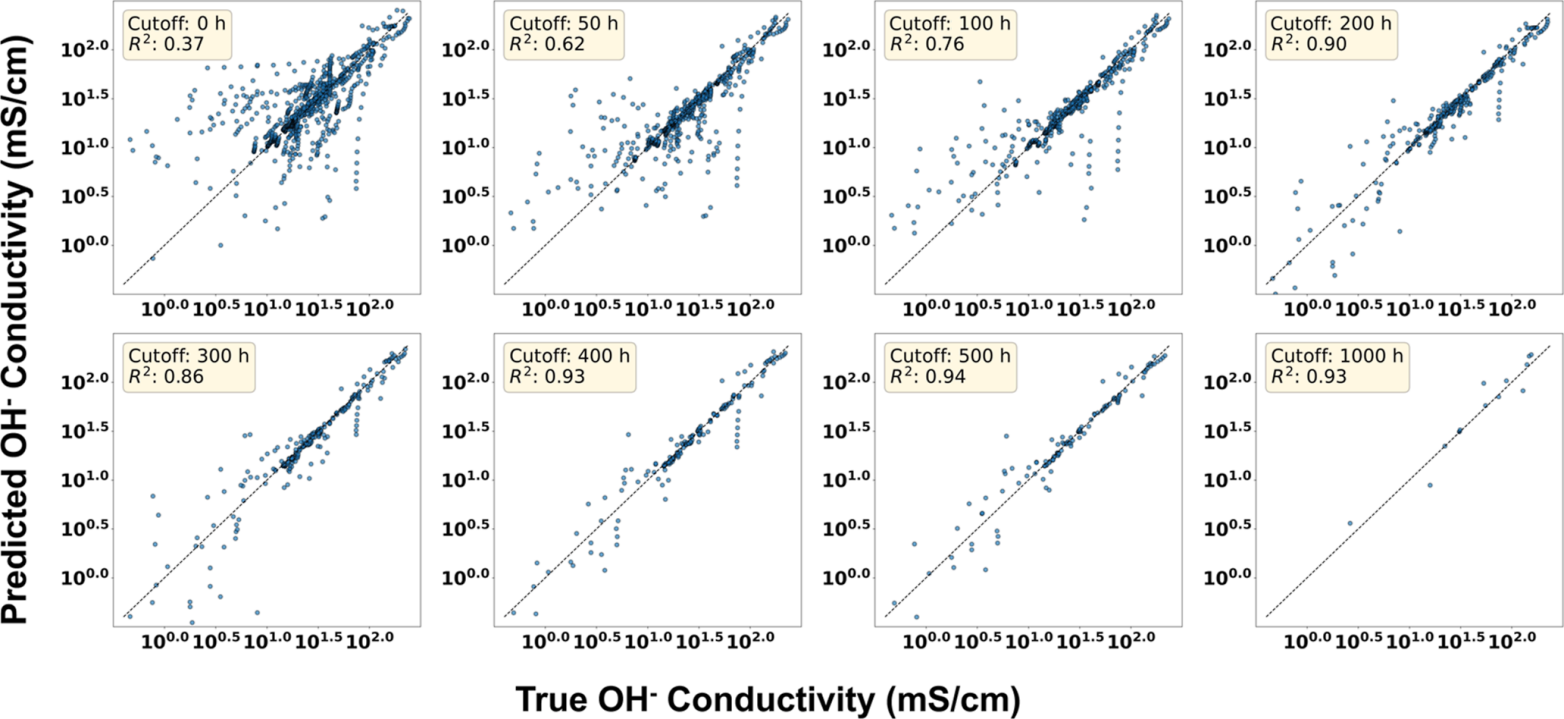
Parity plots of predicted and true hydroxide conductivity for each cutoff value using the PENN models. Models were trained on all available data except the portion of each sample’s data beyond the designated cutoff time (0–1000 h); data from other samples beyond that cutoff remained available for training.

**Figure 7:**
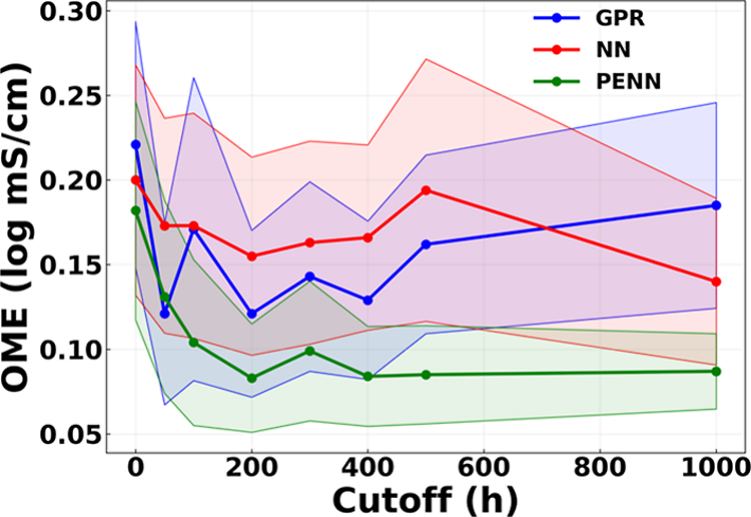
Average order-of-magnitude error (OME) as a function of cutoff time for GPR, NN, and PENN models. Each data point represents the mean prediction error across all test samples withheld from training at a given cutoff. Shaded regions denote ±0.25σ (one-quarter of the standard deviation) around the mean OME, illustrating variability across cutoff values and training algorithms.

An example degradation profile for one sample across each cutoff value for each model is shown in Figure 8. The results enforce the benefit of using physics-based modeling in long-term degradation performance as a cost-saving measure for materials design initiatives. This approach mimics realistic experimental constraints, where extended aging studies may be infeasible due to time, cost, or material limitations. By evaluating model performance at increasing time thresholds, we identify the earliest time point at which partial degradation data becomes predictive of long-term behavior. This analysis provides insight into the temporal data requirements for reliable forecasting and supports the design of efficient experimental protocols. Ultimately, this forecasting capability enables rapid, data-efficient screening of AEM candidates based not only on their initial properties but also on their projected durability.

**Figure 8:**
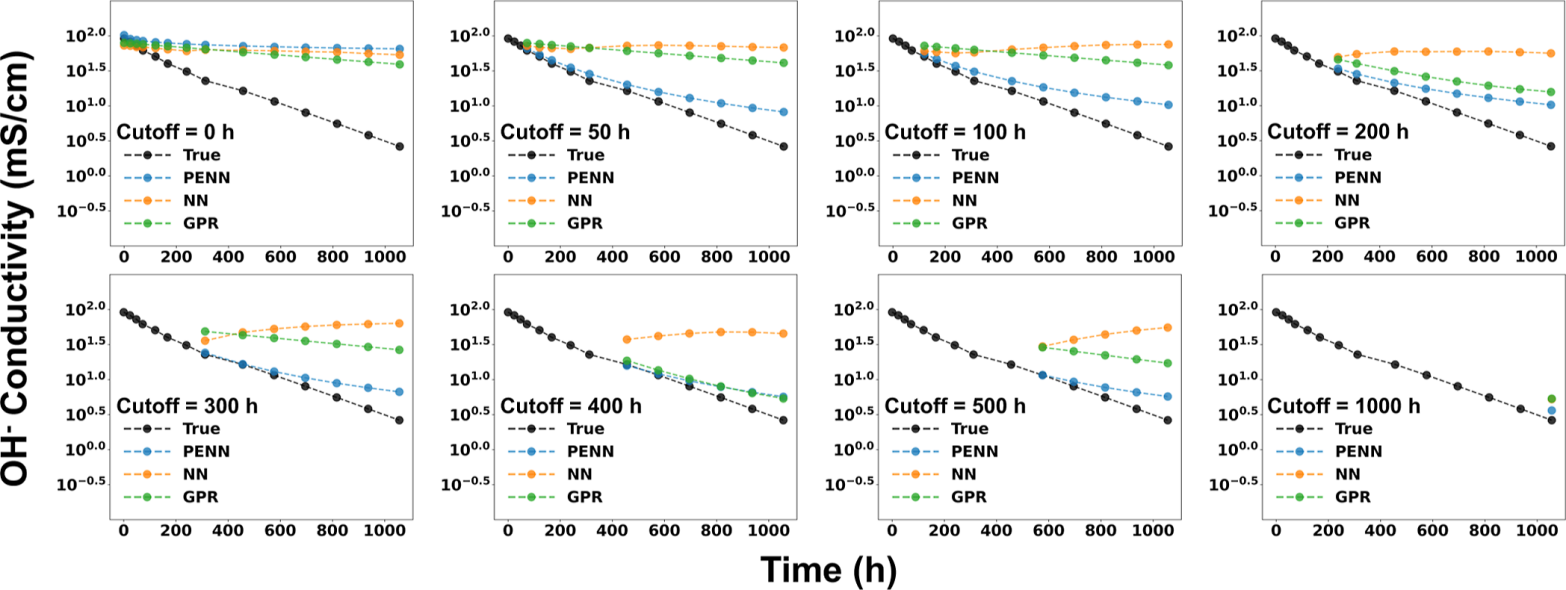
Representative degradation forecasting curves comparing PENN (blue), NN (orange) and GPR (green) predictions against experimental data (black) for a single AEM sample across a range of cutoff values (trained on data for all other samples plus the data up until the cutoff point, and predicted on data after the cutoff point). Predictions using the PENN models drastically improve with small amounts of data, and become more accurate with the inclusion of more data. NN models behave nonphysically (increasing conductivity predictions with time) and GPR models are unable to match the trend predicted by PENN.

### Conclusions, Limitations, and Future Work

We introduced a physics-enforced neural network (PENN) that couples polymer-genome fingerprints and environmental descriptors with a mechanistic degradation equation to model the time evolution of hydroxide conductivity in AEMs. Using a literature-curated data set of time-resolved measurements, PENN (i) learns four interpretable parameters (σ_0_, σ_∞_, *t*_0_, α) that quantify initial performance, long-term limits, time scales, and decay shape; (ii) reveals a normalized universal degradation curve across diverse chemistries and conditions; and (iii) outperforms baseline NN and GPR models in forecasting long-term behavior from sparse early time data. Practically, we find that ∼200 h of measurements often suffices to enable accurate extrapolation toward thousands of hours, reducing experimental burden while preserving physical fidelity. These capabilities position PENN as a data-efficient, interpretable framework for accelerated AEM screening and design based on both initial performance and projected lifetime.

While the PENN framework offers strong predictive performance and interpretability, several limitations should be acknowledged:

- Fixed Functional Form: The degradation model assumes that conductivity decay universally follows eq 1. While this empirically fits the data well, real-world degradation may involve multistage or nonsigmoidal dynamics in some chemistries.
- Chemical Space Limitations: The model generalizes most reliably within the chemical space represented in the training data, and predictions for novel polymer backbones, cations, or additives may carry increased extrapolation uncertainty. Subgroup-specific master curves (e.g., by backbone or cation class) are an interesting future extension but are currently limited by the number of time-resolved samples per subgroup. As the data set size grows, this point may be adequately addressed in the future. Although the present implementation does not include explicit Bayesian or conformal uncertainty quantification, the PENN provides interpretable uncertainty proxies through the dispersion of predicted degradation parameters (σ_0_, σ_∞_, *t*_0_, α), as illustrated in Figure 5, enabling identification of lower-confidence predictions in sparsely populated regions of parameter space. Future work will incorporate explicit out-of-distribution detection and calibrated uncertainty estimates to establish trust boundaries for high-throughput screening and long-term forecasting.
- Single-Property Focus: This work focuses solely on hydroxide conductivity. However, mechanical degradation and dimensional stability are also critical to AEM lifetime. A future extension to multitask PENNs could jointly model conductivity, swelling, and tensile degradation.
- Morphological Descriptors: Hydration structure, ion solvation, and microphase morphology strongly influence hydroxide transport and degradation kinetics in AEMs. In this study, these effects are incorporated implicitly through experimentally accessible scalar descriptors such as relative humidity, solvent concentration, and temperature, which serve as proxies for hydroxide activity and hydration state. While explicit morphology-aware descriptors—such as water-channel connectivity from atomistic simulations or domain spacing from scattering experiments—could further strengthen the chemistry-structure-kinetics linkage, such data are not consistently available in the literature-curated data set. Future hybrid PENN frameworks integrating simulation-derived structural features with experimental data represent a promising direction as multiscale simulation-ML pipelines mature.
- Operando Data: this study focuses on open-circuit chemical aging to isolate intrinsic alkaline stability; however, real fuel-cell operation involves coupled electrochemical stressors such as potential gradients, current density, catalyst–polymer interfaces, and radical or peroxide formation. The PENN framework is environment-agnostic and can be extended by incorporating such variables as additional descriptors. Future work will explore retraining the model on electrochemical or operando data sets to capture field-assisted degradation mechanisms relevant to device operation.
- Experimental Validation: model predictions—especially forecasts beyond 1000 h—should be validated experimentally. PENN provides hypotheses for long-term behavior but should be used as a screening and guidance tool.

Overall, the PENN framework demonstrates superior robustness, physical consistency, and generalizability compared to traditional regression models and neural networks. By integrating domain-specific constraints and leveraging a parametrized degradation equation, it enables accurate modeling across diverse chemical and environmental conditions, identification of universal trends in AEM degradation, quantification of meaningful degradation parameters, and reliable forecasting from sparse experimental data. This modeling framework provides a foundation for accelerated screening of AEM candidates, allowing researchers to prioritize materials based not only on their initial performance but also their projected lifetime.

## References

[1] Dario R. D. (2018). Review
of cell performance in anion exchange membrane fuel cells. J. Power Sources.

[2] Tran H., Gurnani R., Kim C., Pilania G., Kwon Ha-K., Lively R. P., Ramprasad R. (2024). “Design
of functional and
sustainable polymers assisted by artificial intelligence”.
en. Nat. Rev. Mater..

[3] Technical Targets for Proton Exchange Membrane Electrolysis. en. https://www.energy.gov/eere/fuelcells/technical-targets-proton-exchange-membrane-electrolysis. Accessed January 25, 2024. (Visited on 01/25/2024).

[4] Lee W.-H., Kim Yu S., Bae C. (2015). “Robust Hydroxide Ion Conducting
Poly­(biphenyl alkylene)­s for Alkaline Fuel Cell Membranes”. ACS Macro Lett..

[5] Hossen M. M., Hasan M. S., Sardar M. R. I., Haider J. b., Mottakin, Tammeveski K., Atanassov P. (2023). State-of-the-art
and developmental trends in platinum
group metal-free cathode catalyst for anion exchange membrane fuel
cell (AEMFC). Appl. Catal., B.

[6] Merle Géraldine, Wessling M., Nijmeijer K. (2011). Anion exchange
membranes for alkaline
fuel cells: A review. J. Membr. Sci..

[7] Arges C. G., Zhang Le (2018). Anion Exchange Membranes’
Evolution toward High Hydroxide
Ion Conductivity and Alkaline Resiliency. ACS
Appl. Energy Mater..

[8] Mandal M. (2021). “Recent
Advancement on Anion Exchange Membranes for Fuel Cell and Water Electrolysis”. ChemElectroChem.

[9] “Hydrogen and Fuel Cell Technologies Office Multi-Year Program Plan” (2024).

[10] Willdorf-Cohen S., Zhegur-Khais A., Ponce-González J., Bsoul-Haj S., Varcoe J. R., Diesendruck C. E., Dekel D. R. (2023). “Alkaline
Stability of Anion-Exchange Membranes”. ACS Appl. Energy Mater..

[11] Raut A., Fang H., Lin Yu-C., Sprouster D., Yin Y., Fang Y., Fu S., Sharma S., Wang L., Bae C., Rafailovich M. (2023). “Effect of membrane mechanics on AEM fuel cell
performance”. Energy Adv..

[12] Otmi M. Al, Lin P., Schertzer W., Colina C. M., Ramprasad R., Sampath J. (2024). Investigating Correlations in Hydroxide Ion Transport
in Anion Exchange Membranes from Atomistic Molecular Dynamics Simulations. ACS Appl. Polym. Mater..

[13] W., Schertzer , S., Shukla , A., Sose , R., Rafiq , M. Al., Otmi , J., Sampath , R. P., Lively , R., Ramprasad “AI-driven design of fluorine-free polymers for sustainable and high-performance anion exchange membranes”.J. Mater. Inf. 2025, 5, 10.20517/jmi.2024.69.

[14] Tran H., Shen K.-H., Shukla S., Kwon Ha-K., Ramprasad R. (2023). Informatics-Driven
Selection of Polymers for Fuel-Cell Applications. J. Phys. Chem. C.

[15] Mustain W. E., Chatenet M., Page M., Kim Yu S. (2020). “Durability
challenges of anion exchange membrane fuel cells”. Energy Environ. Sci..

[16] Jiang Z., Yi G., Yao X., Ma Y., Su X., Liu Q., Zhang Q. (2023). Durable and highly-efficient anion exchange membrane water electrolysis
using poly­(biphenyl alkylene) membrane. Chem.
Eng. J..

[17] Song W., Peng K., Xu W., Liu X., Zhang H., Liang X., Ye B., Zhang H., Yang Z., Wu L., Ge X., Xu T. (2023). “Upscaled
production of an
ultramicroporous anion-exchange membrane enables long-term operation
in electrochemical energy devices”. Nat.
Commun..

[18] Batra R., Song Le, Ramprasad R. (2020). “Emerging
materials intelligence
ecosystems propelled by machine learning”. Nat. Rev. Mater..

[19] Nistane J., Datta R., Lee Y. J., Sahu H., Jang S. S., Lively R., Ramprasad R. (2025). “Polymer
design for solvent
separations by integrating simulations, experiments and known physics
via machine learning”. npj Comput. Mater..

[20] Kim C., Chandrasekaran A., Huan T. D., Das D., Ramprasad R. (2018). Polymer Genome:
A Data-Powered Polymer Informatics Platform for Property Predictions. J. Phys. Chem. C.

[21] automeris.io: Computer vision assisted data extraction from charts using WebPlotDigitizer. https://automeris.io/Accessed June 6, 2025.

[22] C., Kuenneth , W., Schertzer ; R., Ramprasad “Copolymer Informatics with Multitask Deep Neural Networks”. In: Macromolecules 54.13 (July 2021). Publisher: American Chemical Society, pp 5957–5961. issn: 0024–9297. doi: 10.1021/acs.macromol.1c00728.

[23] Zou X., Pan Ji, Sun Z., Wang B., Jin Z., Xu G., Yan F. (2021). “Machine
learning analysis and prediction models of alkaline
anion exchange membranes for fuel cells”. Energy Environ. Sci..

[24] Yin, K. P. , T., Fujigaya , K., Kato “Predicting the anion conductivities and alkaline stabilities of anion conducting membrane polymeric materials: development of explainable machine learning models”. In: Science and Technology of Advanced Materials 24.1 (Dec. 2023). Publisher: Taylor & Francis _eprint: DOI: 10.1080/14686996.2023.2261833, p 2261833. 37854121 10.1080/14686996.2023.2261833 PMC10580864

[25] Pedregosa F., Varoquaux G.¨l., Gramfort A., Vincent M., Bertrand T., Grisel O., Blondel M., Prettenhofer P., Weiss R., Vincent D., Vanderplas J., Passos A., Cournapeau D., Brucher M., Perrot M., Duchesnay E. ´douard (2011). Scikit-learn: Machine Learning in
Python. J. Mach. Learn. Res..

[26] Paszke, A. ; Gross, S. ; Massa, F. ; Lerer, A. ; Bradbury, J. ; Chanan, G. ; Killeen, T. ; Lin, Z. ; Gimelshein, N. ; Antiga, L. ; Desmaison, A. ; Kopf, A. ; Yang, E. ; DeVito, Z. ; Raison, M. ; Tejani, A. ; Chilamkurthy, S. ; Steiner, B. ; Fang, Lu. ; Bai, J. ; Chintala, S. “PyTorch: An Imperative Style, High-Performance Deep Learning Library”. In Advances in Neural Information Processing Systems; Curran Associates, Inc., 2019; Vol. 32.

[27] Akiba, T. , Sano, S. , Yanase, T. , Ohta, T. , Koyama, M. “Optuna: A Next-generation Hyperparameter Optimization Framework”. In: Proceedings of the 25th ACM SIGKDD International Conference on Knowledge Discovery & Data Mining. KDD ’19. Association for Computing Machinery, New York, NY, USA, July 2019; pp 2623–2631.

